# Gender and Stand Your Ground Laws: A Critical Appraisal of Existing Research

**DOI:** 10.1017/jme.2023.40

**Published:** 2023

**Authors:** Caroline Light, Janae Thomas, Alexa Yakubovich

**Affiliations:** 1: HARVARD UNIVERSITY, CAMBRIDGE, MA, USA; 2: QUINTAIROS, PRIETO, WOOD, AND BOYER, TAMPA, FL, USA; 3: DALHOUSIE UNIVERSITY, HALIFAX, NS, CANADA

**Keywords:** Stand Your Ground, Intersectionality, Gender, Domestic Violence, Intimate Partner Violence, Battered Woman Syndrome

## Abstract

This paper evaluates the existing research on Stand Your Ground (SYG) laws in terms of the extent to which it has accounted for gender. In particular, we address (a) what the available evidence suggests are the gender-based impacts of SYG laws and (b) where, how, and why considerations of gender may be missing in available studies.

While there are numerous studies that address the racial implications of “Stand Your Ground” (SYG) laws, there are comparatively few that consider gender.^1^ One result of this disparity is a research terrain that downplays or ignores how the expansion of justifiable homicide — a legal principle that has historically privileged already empowered social actors — reinforces gender injustice. For instance, promoters of SYG laws emphasize the need to protect citizens from threats outside the home, while ignoring the fact that intimate partner violence (IPV) and, more broadly, domestic violence (DV), have been and remain the most common forms of violence against women.^2^ This contradiction — between actual versus alleged threats of gender violence — rests at the heart of contemporary ignorance of the impact SYG laws have on women, especially those who experience multiple forms of social subordination, such as racism, heterosexism, ableism, and class inequity.

In this paper, we evaluate the existing research, through 2020, on SYG laws to determine the extent to which gender is considered. We address (a) what the available evidence suggests are the gender-based impacts of SYG laws, (b) where, how, and why considerations of gender may be missing in available studies, and (c) what an intersectional approach to gender contributes to SYG related scholarship. The essay proceeds in four parts. First, we provide a summary of SYG laws. Second, we offer a critical review of existing scholarship on the gender implications of SYG laws, separated into a focus on empirical quantitative and qualitative studies as well as legal, philosophical, sociological, and historical work. Third, we identify gaps around *intersectional* analysis, which considers the simultaneity of white supremacy, classism, and other power structures that influence the way gender is interpreted and experienced. We conclude that an intersectional approach is essential for understanding and evaluating contemporary laws that expand civilian rights to use deadly force in self-defense. We outline our recommendations for more rigorous, intersectional gender analysis of SYG laws and the impacts of these laws on existing socio-legal inequities.

## Stand Your Ground Laws and the Evolution of “Self-Defense”

SYG laws are amendments to existing “Justifiable Use of Force” statutes. First introduced in Florida in 2005, they expand the geospatial and situational terms under which a person may legally use force in self-defense, by abolishing the traditional duty to retreat when a person reasonably believes they face an imminent threat of death or serious bodily harm. SYG laws draw upon the logic of the “Castle Doctrine,” which authorizes the use of force, without a duty to retreat, when one is threatened in one’s home. However, the doctrine often does not apply to co-habitants, who are expected to try to retreat before using force in self-defense.^3^ SYG laws expand the boundaries of the “castle” by allowing the use of deadly force in any space where a person is legally entitled to be, even when retreat is safely possible.^4^ While all 50 states have implemented some version of the Castle Doctrine, as of fall 2022, thirty states have removed the duty to retreat through written statutes. An additional eight states apply the change in justifiable use of force laws through case law and jury instructions, which effectively function as SYG laws, removing the duty to retreat wherever a person may legally be.^5^

In other words, SYG laws provide a more seamless pathway out of legal jeopardy for those who claim to have stood their ground in protection of property as well as human life, and alleged self-defenders may avoid arrest, court, or any legal charges, given that law enforcement can be held liable for arresting those who are found to have reasonably acted in self-defense.

Supporters of SYG laws claim they fortify public safety by enabling “law-abiding citizens” to defend themselves from dangerous criminals. Indeed, Jeannie Suk Gerson suggests that the laws’ main accomplishment is to remove the burden of proof from the claimant/self-defender, who no longer needs to prove that they were “reasonably” fearful of harm or death at the moment when they used deadly force against a perceived perpetrator.^6^ Other legal scholars, including Donna Coker and Mary Anne Franks, have argued that the traditional laws governing justifiable homicide already protected those who lawfully used force in self-defense when no retreat or escape was possible. Depending on the state in which the encounter took place, courts would weigh the evidence and determine whether a claimant’s use of lethal force was justifiable. Prior to the establishment of SYG laws, a person attacked outside of their home would not be held liable if they could prove that their use of force was their only means of escape from a deadly threat or serious bodily injury.^7^ For this reason, some critics of SYG laws, including Joe Grace, Executive Director of CeaseFirePA and criminologist John Roman, have characterized them as “a solution in search of a problem.”^8^

While the original laws governing self-defensive force already provided significant protections for some civilians, SYG laws created additional protections in the form of: (1) immunity from criminal prosecution and civil action for claimants found to have reasonably “stood their ground” after using lethal force and (2) the capacity to use lethal force against someone in the act of committing a “forcible felony,” which includes property theft or destruction.^9^ In other words, SYG laws provide a more seamless pathway out of legal jeopardy for those who claim to have stood their ground in protection of property as well as human life, and alleged self-defenders may avoid arrest, court, or any legal charges, given that law enforcement can be held liable for arresting those who are found to have reasonably acted in self-defense.^10^

Moreover, some states have modified SYG laws to provide additional legal protections for those who claim to have used force in self-defense. In 2010, Florida instituted a pre-trial evidentiary hearing, which provided claimants with an early opportunity to obtain immunity from further prosecution.^11^ By 2017, the state legislature had removed the burden of proof from the defense and placed it on the prosecution.^12^ This revision has made it increasingly difficult to convict an individual who claims self-defense, especially if the only other witness to the encounter is deceased.

Although the race- and gender-neutral language of SYG laws suggests that they apply universally, the removal of the duty to retreat has been shown to create distressing consequences for those who experience various forms of structural subordination, particularly racism. Contrary to proponents’ claims that SYG laws prevent crime and empower would-be crime victims, a 2015 American Bar Association task force found that the laws exacerbate violence and existing racial biases in the criminal justice system.^13^ According to Jacinta M. Gau, Kareem L. Jordan, and Katheryn Russell-Brown, SYG laws contribute to the characterization of Black and other non-white people, particularly men and boys, as “reasonable threats,” especially when an alleged self-defender is white.^14^ Recent criminological and epidemiological research reveals that white people who kill Black people and claim self-defense are significantly more likely to be exonerated or to escape prosecution entirely in states with SYG laws.^15^ Based on the convincing empirical evidence that states with SYG laws experience increased homicide rates in addition to amplifying racial biases in the law, the 2015 ABA task force recommended that the laws be repealed or seriously amended.^16^

While there is abundant research on the ways SYG laws exacerbate existing racial inequalities, very little scholarship — empirical or otherwise — accounts for gender. Further, the few studies that address the gender implications of SYG laws often fail to account for the simultaneity of race and gender inequities in SYG laws and traditional self-defense laws more generally. One exception is Terressa Benz’s sociological analysis of the 2017 case of Siwatu-Salama Ra, a pregnant Black woman in Michigan who was charged with felonious assault with a firearm after brandishing an unloaded gun at driver who was threatening her family. Beyond Benz’s detailed investigation of the “controlling images” that “cast Black women as domineering, aggressive, irresponsible mothers, who are incapable of the fear necessary to invoke SYG,” most studies lack a robustly *intersectional* analysis that considers how gender, race, class, and other categories of identity intersect to exclude most women, particularly women of color, from the purported benefits of SYG laws.^17^ Given that Black women and other women of color are disproportionately at risk in the U.S. for violence and criminalization, we urge that ongoing studies of SYG laws take pains to place them at the center of their analyses.^18^ Otherwise, we risk obfuscating the complex socio-legal, historic, and epistemic effects of SYG laws, particularly their distorting impact on the nation’s criminal legal system. In the following sections, we summarize knowledge gaps across the empirical and legal analyses and recommend areas for methodological improvement in future research.

## Empirical Evaluations of Stand Your Ground Laws

In 2021, Yakubovich et al. conducted a systematic review of quantitative research on the impacts of SYG laws on violence, injury, firearm, and criminal justice outcomes. The study examined whether there are different impacts based on sociodemographic characteristics, including by race or gender.^19^ The review showed that SYG laws dramatically increased homicide rates in some states and have been associated with racial inequities in criminal justice outcomes. However, the study found little evidence of gender-based analysis in the field and a lack of focus on the impacts of SYG laws on IPV (violence between romantic partners) or DV (violence that takes place within a household or family), the most common forms of violence against women. Most quantitative studies estimating SYG laws’ effects have not addressed differences in the rates or judicial processing of cases involving DV or IPV. The studies also fail to address the unique burdens of violence experienced by Black women at the intersections of racism and sexism, including systemic biases in institutional responses.^20^

One notable exception was a study conducted by Justin Murphy, which investigated differences in the outcomes of 237 SYG cases in Florida recorded in the *Tampa Bay Times* from 2005 to 2013 by the race, sex, and relationship of the victim and claimant (i.e., the person claiming self-defense).^21^ Murphy’s was the only available study that conducted interaction analyses and considered all possible pairwise interactions between the race and gender of claimants and victims as well as the correlation between DV and gender. Cases where the victim was white had higher odds of ending in conviction when the claimant was a racial minority or the victim was female. Murphy further found that cases involving DV had higher conviction rates when the claimant was female. Importantly, these analyses were adjusted for other potential variables, such as whether there was a firearm involved or the claimant could safely retreat. These initial results, while limited to a single study in Florida, suggest that there are racial and gendered biases in the application of SYG laws in legal decision-making that warrant further investigation. Murphy’s sample size was too small to explore the differences in odds of conviction by the three-way interaction of race, gender, and relationship type (domestic versus non-domestic).

An additional descriptive study by Denise Crisafi, which did not estimate associations, found that more men than women were convicted in 57 SYG cases that involved IPV across states.^22^ This finding stands in contrast to Murphy’s results. Possible reasons for discrepancies may be that the Crisafi study (1) only conducted cross-tabulations (and did not adjust for other variables); (2) focused specifically on IPV rather than DV broadly; and (3) used a different sample of cases beyond Florida. However, Crisafi noted that, among those found guilty, women tended to face longer sentences than men, suggesting another gender-based bias against women in the judicial application of SYG laws.^23^ Maeve O’Brien offered a potential explanation for these results, identifying four main challenges that women who killed their abusers faced in convincing judges and juries that they had acted in self-defense. These included: (1) a lack of documentation (e.g. police reports) proving their past experience of abuse; (2) a general perception that abusive men are non-deadly as long as they are unarmed; (3) prosecutors’ tendency to portray female defendants as liars with ulterior motives, rather than as victims of abuse; and (4) courts’ misunderstanding and misinterpretation of expert testimony relating to IPV and the gender dynamics of relationship violence.^24^ Notably, there were only ten eligible cases for analysis from the total sample of 234 cases, and O’Brien did not consider other variables, including race, religion, or jury composition.^25^

## Legal Limitations of an Equitable Right to Self-Defense

While the empirical studies provide a vital lens on the existence of inequities in the adjudication of SYG laws, the patterns of gender bias differ across the available studies. Further, the explanations are limited due to methodological variations and small sample sizes restricted to certain jurisdictions (e.g., Florida) and time periods. Alongside the empirical research, (socio)legal and historical analyses of gender and SYG laws provide additional insights on the ways traditional and contemporary laws place women at a disadvantage when they use self-defensive violence, especially against men they know. In contrast to studies exploring the racial implications of SYG laws, which tend to focus on violence between strangers, most of the gender-related analyses address IPV, DV, or a combination of the two.

A key contribution of the legal scholarship is to highlight the gender exclusions embedded in traditional self-defense laws which have, in turn, influenced contemporary SYG laws. Analyses by Coker, Franks, Gillis, Keegan, Messerschmidt, and Suk expose how gendered tropes of violence and vulnerability underpin traditional self-defense laws, with dismal consequences for women who try to defend themselves from abusers with whom they cohabit.^26^ Suk was first to address the gender implications of the “new Castle Doctrine laws,” establishing the vital linkage between gendered ideals of home and the evolution of laws governing civilian use of force in self-defense.^27^ She tracked how the idealized figure of the “True Man” has been empowered over time to use lethal force in self-defense wherever he has the right to be. Suk’s analysis of the “True Man,” who is without fault in “resisting force with force,” has since been taken up by other feminist scholars to illuminate the gender double standard by which courts have traditionally adjudicated cases of DV or IPV related homicides, and the subsequent failure of SYG laws to grant abused women a legal means to defend themselves from their abusers.

The legal scholarship critically addresses the use of “Battered Woman Syndrome” (BWS) as an innovation to accommodate the particular circumstances of the victim of DV or IPV who uses force against her abuser, particularly when the abuser is not posing what many would consider an “imminent” threat. For instance, women often kill their abusers when they are sleeping or otherwise unarmed or incapacitated, rather than in the throes of a violent confrontation. In the late 1970s, psychologist Lenore Walker introduced BWS as a psychological theory of abused women’s mindset and behavior in the context of a cycle of violence inflicted by her intimate partner. The theory explains why patterns of cyclical violence may cause victims to adopt behavioral patterns that do not seem “rational” to outside observers, such as “learned helplessness,” where an abused woman feels trapped, without any options or autonomy.^28^ Defense attorneys use BWS to help juries understand abuse victims’ behaviors, such as killing an abuser while he is unarmed or sleeping. However, as a defense strategy, BWS not only reinforces gender stereotypes of abused women’s intrinsic irrationality, its success depends upon the defendant’s ability to emulate characteristics associated with feminine vulnerability, such as being “weak, passive, and fearful.”^29^ In stark contrast to the virtuous violence of the “True Man,” who heroically stands his ground wherever he may legally be, the morally and legally suspect “Battered Woman” is pathologized as weak and irrational. Furthermore, she depends on expert testimony to prove her psychological condition of learned helplessness.^30^

The legal scholarship illuminates the gendered dimensions of the practical differences between SYG and BWS. Franks explains that while SYG is a “justification defense” — a defense based on a person’s having conducted themselves lawfully — BWS is perceived as “an excuse defense, implying that the defendant’s wrongful behavior might be legally excused by her ‘irrational’ state of mind.”^31^ While a successful SYG claimant has performed heroically in their deployment of rightful violence, a BWS claimant, according to Franks, “plead[s] for mercy on the basis of what is essentially considered a psychological defect.”^32^ Crucially, the narrow requirements of feminine victimhood — helplessness, passivity, and sexual chastity — disproportionately exclude the most vulnerable types of abuse victims, including LGBT+ and low-income women and women of color.^33^ However, with the exception of studies by Benz, Franks, Gillis, and Coker, the legal research on SYG laws and gender has not accounted for race, sexuality, ethnicity, or class.

Considering identities beyond gender allows us to see through what might appear on the surface as a “feminist” solution to gender violence. While SYG supporters exhort women to purchase and carry handguns and to “stand their ground” against their attackers, Franks argues that the laws were never intended to provide women a legal means to resist violence from their own intimate partners and ex-partners.^34^ Most SYG statutes fail to address situations in which the claimant and her victim/assailant share the same home. Those states with SYG laws that do acknowledge relationship violence typically only remove the duty to retreat if the claimant has an active protection order, which can be difficult to obtain.^35^ As Allard, Brown, Crenshaw, Crisafi, Gillis, and Richie have illustrated, there are significant structural barriers to obtaining protective orders, especially for non-white and low-income women.^36^

The scholarship under review shows how legal standards of “reasonableness” and “imminence” are gendered in ways that exclude abuse survivors from legal pathways to exoneration after using force against their abusers. Traditional self-defense laws were based upon masculine assumptions of “reasonableness” that assume that the most urgent threats are sudden attacks from strangers or home intruders. Such assumptions, alongside historic beliefs in husbands’ chivalric protection of their wives, obfuscate the reality of longstanding threats from within the household itself. Since most women’s self-defensive behavior is against men they know, *not* threatening strangers, neither SYG nor traditional self-defense laws have provided them with reliable legal protection. A focus on gender complicates the law’s “imminence” requirement that the person using force in self-defense must not have started the altercation, because it does not take into consideration long-term patterns of abuse. Many women who kill their abusers do so in “nonconfrontational circumstances,” when their abuser is not posing an “imminent” threat. Even if they kill in response to a pattern of past abuse that has led them to perceive their partner as a deadly threat, courts are likely to see such claimants as having initiated the lethal confrontation.^37^

The doctrine of equal or commensurate force similarly disadvantages women whose abusers tend to be physically larger and stronger. One result, according to Franks, is that SYG laws offer “reassurance and encouragement to men who would not only initiate violent encounters with strangers in public places, but also those who attack their wives in the privacy of their own homes. It reinforces a quasi-right for men to advance far from their homes to start fights, and a quasi-duty for women to retreat from their own homes instead of fighting back.”^38^ Indeed, the legal scholarship shows how SYG law “solidifies gender stereotypes” by reinforcing the “empowerment doctrine of the true man and the helplessness ideology behind BWS.”^39^ Benz, Coker, Franks, and Gillis emphasize how these gender stereotypes interact with racism and other structurally embedded systems of injustice, resulting in unequal adjudication of SYG laws.^40^

With the exception of Gillis, who discusses Maryland laws and cases from several different states, and Benz, who analyzes the 2017 case of Siwatu-Salama Ra in Michigan, most of the criminological or legal scholarship focuses on Florida. Coker, Franks, and Abuznaid discuss the Florida case of Marissa Alexander to highlight the intersectional implications of SYG laws.^41^ Alexander, a licensed gun owner, fired a warning shot to escape her abusive, estranged husband in 2010, only to be prosecuted for aggravated assault with a lethal weapon. In stark contrast to the case of George Zimmerman, who initiated a lethal confrontation with unarmed 17-year-old Trayvon Martin, Alexander was depicted as an “angry” perpetrator of violence despite her well-documented history of experiencing abuse at the hands of her spouse.^42^

As we interrogate the legal structures and processes as they apply to women who fight back against their abusers, we must also unpack the racial implications of the “True Man” doctrine. While some acknowledge the presumption that the “True Man,” who is “without fault,” is most often a white property-owner, Messerschmidt takes the analysis further. She attends to the ways in which self-defense laws have transformed over the twentieth and twenty-first centuries to elevate property rights over the rights of people to live free from violence and abuse.^43^ And while her study does not consider the impact of race or class specifically, her critique of SYG law’s destructive focus on property interests is essential to understanding how lawmakers and promoters of SYG laws — and their recent offspring, “anti-rioting” legislation — enshrine an implicitly gendered and racialized right of the “True Man” to use lethal violence in the protection of property.^44^

Much of this lethal violence involves guns, to which Jennifer Carlson and Kristin Goss attend in their analysis of the rapid proliferation of firearms as “everyday objects” affecting the spread and impact of SYG laws. Their study provides a wider historical analysis of the way government regulation of firearms both manages and emulates gendered ideals and behaviors. They also present gender as “a powerful theoretical lens” with which to interrogate the Second Amendment.^45^ Of the three chronological governance strategies the authors explore, “fixed governance” coincides with the contemporary spread of SYG laws, in which guns are perceived as essential for self-defense.^46^

The studies under review indicate that there are important ways that the justice system disadvantages women — especially women of color and low-income women — who invoke SYG laws, and especially in the context of IPV and DV. They point to an urgent need for further research that examines the outcomes of SYG cases by gender, race, and their intersection across states, with attention to different forms of domestic violence, and that accounts for variations in case characteristics and state laws.

Citing Judith Butler, Carlson and Goss envision “gender [as] the social laid upon the biological,” which allows for an interrogation of the nuances of gendered patterns of governance concerning firearms.^47^ Their discussion of self-defense generally, and SYG laws specifically, interrogates the state’s efficacy in protecting women from threats outside the home. In concert with the legal literature, their study addresses how SYG laws emphasize stranger violence to the exclusion of violence between acquaintances. While their study constitutes a comprehensive historical review of regulatory structures and federal lawmaking on guns, the historical patterns of racialized gender that implicate stranger violence and “good citizenship” as a trope of racialized masculinity would be a valuable addition.

The legal and criminological studies provide several proposals for reforming the unequal contemporary self-defense terrain. Given that the Castle Doctrine and most SYG laws do not remove the duty to retreat for cohabitants, Suk, Messerschmidt, and Gillis propose that the Castle Doctrine be applied in jury instructions when a defendant shared her “castle” with the person against whom she used self-defensive force. Per Suk, removing the duty to retreat in the household would provide abused women the opportunity to “stand their ground against their batterers.”^48^ Given the gender exclusionary realities of the “reasonability” and “imminence” requirements, Gillis and Keegan recommend the creation of a self-defense presumption unique to women who have experienced DV. Keegan proposes a “new legal framework of individualization,” obligating courts to consider the complex, specific experiences and conditions under which a defendant resorted to violence.^49^ Gillis proposes a legal presumption of self-defense that would apply to cases involving a woman killing a male family member or intimate partner, alongside a pre-trial presumptive self-defense hearing, and a presumption of self-defense that the prosecution must disprove beyond a reasonable doubt.^50^ While Gillis claims these innovations – given their narrow application in DV or IPV cases – would not increase racially motivated killings, there are other race and class considerations, such as the unique ways in which women of color and low-income women may be subject to additional modes of criminalization that may undermine their access to a pre-trial hearing.

The studies under review indicate that there are important ways that the justice system disadvantages women — especially women of color and low-income women — who invoke SYG laws, and especially in the context of IPV and DV. They point to an urgent need for further research that examines the outcomes of SYG cases by gender, race, and their intersection across states, with attention to different forms of domestic violence, and that accounts for variations in case characteristics and state laws. Further, although some studies acknowledge the role of white supremacy in shaping the legal architectures of self-defense, future research on SYG laws would benefit from a robust integration of feminist intersectional methods to amplify the complex plight of DV and IPV survivors who are excluded from the right to defend themselves.

## The Need for Intersectional Analysis

While many of the studies surveyed address DV and/or IPV, we note a widespread inadequacy in accounting for the experiences of minority women in self-defense cases and SYG more specifically. With the exception of Benz, most studies that focus on gender position the experiences of white women as the default although Black women are more likely to be targets of violence from strangers as well as from intimate partners.^51^ Black women are 2.5 times more likely than white women to experience physical or sexual violence from a partner or spouse, and they are also more likely to lack access to mental and physical health services.^52^ According to the 2011 National Intimate Partner and Sexual Violence Survey, approximately 41% of Black women have experienced physical violence by an intimate partner during their lifetime, compared to 31% of white women, 30% of Hispanic women and 15% of Asian or Pacific Islander women.^53^ Due to a complex combination of widespread stigma, experiences of racism, and historical oppression, Black women are less likely to seek help from law enforcement and other formal channels, compared to white women and women of other ethnic and racial backgrounds.^54^ Further, clinical psychologist Jennifer Gómez has traced the complex pressures on Black women — including an effort to avoid further stigmatizing Black masculinity — not to report assaults by a Black men.^55^

Kimberlé Crenshaw’s now-canonical essay “Mapping the Margins: Intersectionality, Identity Politics, and Violence Against Women of Color,” reveals how a unitary focus on *either* racism or sexism “relegate[s] the identity of women of color to a location that resists telling.”^56^ Crenshaw’s critique of traditional identity politics draws from decades of Black feminist advocacy and scholarship to produce a theory of “intersectional” praxis, a way of amplifying the unique experiences of Black women and women of color. Sharon Allard takes up the call for a deliberately intersectional approach in her critique of BWS’s reliance on “prevailing gender characterizations of dominant, white society.”^57^ In her analysis of (1) the historical-legal treatment of Black versus white women and (2) the spurious tropes of Black femininity in U.S. culture, Allard concludes that “gender-based theories that do not incorporate race and class will be as problematic as battered woman syndrome.”^58^ Although Crenshaw’s and Allard’s work appeared before the passage of SYG laws, they offer useful models of Black feminist and intersectional methods of addressing structural injustice. Tracking the “intersecting patterns of racism and sexism” as they affect the lives and choices of non-white women — and women minoritized via other characteristics such as class, (dis)ability, and nationality — is essential to the efficacy of on-going research on self-defense, specifically SYG laws in the context of a burgeoning “gun culture.”^59^

Benz illuminates the exclusionary valences of self-defensive firearm use by placing Black femininity at the center of her analysis. Her close socio-legal analysis of Siwatu-Salama Ra’s case provides an example of the way Black feminist critical methodologies can reveal multiple sites of legal jeopardy experienced by Black women for whom the immunities of SYG laws prove elusive.^60^ Crisafi’s study includes Black, Latinx, Asian American, and Native American as well as white women, considering race and ethnicity as factors influencing women’s experiences of abuse and in their efforts to claim SYG in the context of DV or IPV. Abuznaid et al. explore how “deep-seated gender bias further complicates the status of women of color in American society who exist at the intersection of at least two marginalized groups with respect to race and gender.”^61^ Murphy, citing Crenshaw, recommends further investigation of homicide data to illuminate how “race and gender interact in complicated ways specifically within cases of domestic violence.”^62^ He suggests that the gender inequities of SYG are perhaps even “more pronounced” in his dataset than the racial inequities.

This point underscores the need to examine the extent to which Black women’s experiences with SYG in cases of domestic violence diverge from white women’s, a question that requires a larger dataset of cases to analyze. As Yakubovich et al. highlight, Murphy’s study demonstrated a deliberately “intersectional” quantitative approach, while all other extant studies accounted only for the effects of race and gender on SYG adjudications, and Murphy’s was the only quantitative study to consider intersections between gender and domestic violence. However, all available quantitative analyses of SYG laws, including Murphy’s, are limited to Florida. At present, researchers’ ability to examine these more nuanced questions that require larger sample sizes is limited by the lack of a dataset of self-defense cases beyond Florida and across the U.S.

Most studies we reviewed focus on women, which is justified by the dearth of research on how state criminal law purports to “protect” women even while imposing limitations on their capacity to defend themselves from their abusers. However, to be a truly useful “category of analysis,” gender should apply to social and cultural construction and deployment of masculinities as well as femininities.^63^ Much of the existing scholarship provides robust analysis of the disproportionate masculinity of homicide and violence more generally, unpacking the significance of the “True Man” doctrine as an underlying framework structuring SYG laws. While Goss and Carlson analyze the masculine framing of governance structures, as well as the way the state helps script gender practices and norms, few of the studies under review address the manifold ways in which non-white masculinities are differently gendered from white ones, and how self-defense laws and epistemes of vulnerability and threat presume a non-white figure of predatory masculine “stranger danger.” Studies of SYG laws must address how perceptions of what constitutes a “reasonable” threat rest on implicitly gendered biases against non-white masculinities, and how historic patterns of criminalization position Black men and boys as legitimate or “reasonable” targets of professed self-defensive violence.^64^ Abuznaid et al.’s comparison of the Dunn, Zimmerman, and Alexander cases, which invoked SYG principles with divergent outcomes, reveals the “biases inherent in the criminal justice system that are exacerbated in the uneven application of SYG laws based on the race, age, and gender of the defendant and victim.”^65^

Black feminist scholars, including but not limited to Sharon Allard, Terressa Benz, Patricia Hill Collins, Kimberlé Crenshaw, Angela Davis, Sarah Haley, bell hooks, Jennifer Nash, Beth Richie, Patricia A. Williams, Dorothy Roberts, and Shatema Threadcraft have shown how women of color, especially Black women, have experienced gendered oppression, violence, and criminalization in significantly different ways than white or white adjacent women and Black men.^66^ As Benz writes, “Black women have long been excluded from dominant perceptions of vulnerability,” with severe consequences when they try to invoke self-defense laws.^67^ Historian Sarah Haley revealed how Jim Crow segregation and racial capitalism shaped the unique experiences of incarcerated Black women in the twentieth century South. Her scholarship exposed how “the alleged benefits of the ideology of femininity did not accrue to” Black women charged with crimes in Georgia.^68^ These historic harms persist into the present, and studies of SYG that fail to consider gender alongside race (and class) risk obscuring the “sexual abuse to prison pipeline” by which the legal system criminalizes victims of sexual assault and human trafficking, particularly when they are women and girls of color.^69^ Black girls constitute 14% of the general population nationally but 33.2% of girls detained and committed into juvenile justice detention centers.^70^ Unless our research considers the crosscutting power structures that shape individuals’ experiences of gender and sexual violence, it risks reproducing conceptual and data gaps that in turn reinforce epistemic harms against the most vulnerable populations. Future scholarship on SYG laws must consider the variable ways in which minoritized femininities and masculinities are experienced given our contemporary criminalizing discourses of vulnerability and threat.

## Echoes of the Past: American Self-Defense in Historical Context

The dearth of intersectional analysis makes gender appear unitary and autonomous, while downplaying the role of race in state and individual appeals to “protection” as justification for violence. Carlson and Goss discuss the state’s retreat from the home via a turn to individual armed self-defense against threatening strangers. Yet their analysis overlooks the historical recurrence of a “stranger danger” trope as justification for armed, white supremacist violence, as in settler colonial “Indian wars” or “Savage wars” and armed slave patrols, which in turn laid the ideological groundwork for our contemporary “gun culture.”^71^ As we track the erosion of the duty to retreat, in favor of a “True Man’s” right to “stand his ground,” we must also attend to the exceptions embedded in this expanding right to kill in self-defense. For the “True Man” doctrine excluded not only women — who were legally subordinate to their husbands — but also Indigenous and enslaved people and their descendants. Exclusion from the “True Man” doctrine has sustained a legal legacy under which non-white men, Black men in particular, are not able to access the protections and immunities of the laws governing self-defense, including SYG laws.^72^ While the existing scholarship on SYG laws provides a lens on the law’s complex gender exclusions, future research must consider the indelible marks of white supremacist violence and settler colonialism on traditional self-defense laws and their contemporary offspring.

More research is needed to demonstrate how historic and sociolegal processes produced a contemporary “gun culture” in which appeals to self-defense license a selective right to kill.^73^ Research that attempts to explain the gender exclusions embedded in SYG laws must address the intersecting power structures that produce uneven patterns of regulation around gun ownership and use across gender as well as class, race, and region. Black feminist scholars who have studied lynching, from Ida B. Wells to Crystal Feimster, have noted the exceptional role of gender and sexuality in naturalizing extra-legal violence in the form of “lynch law,” not only in the Deep South, but nationwide.^74^ Nearly 200 anti-lynching bills were brought before Congress between 1890 and 1952, only to be rejected by white male law-makers who claimed that lynching was necessary to protect white women from Black sexual predators.

Central to this logic was (1) the elevation of white women’s sexual purity as sacrosanct and in need of violent protection, alongside (2) the denial that women were most likely to be harmed by their own husbands and family members. Furthermore, the mythological “Black Beast Rapist” or “criminalblackman” had its counterpart in the trope of the sexually aggressive Black “Jezebel,” which helped justify white men’s quotidian sexual violence against Black women and girls.^75^ These gendered and racialized sexual mythologies cloaked white supremacist violence under appeals to white women’s vulnerability —indeed, coding femininity itself *as* white — and lynching took shape as a manifestation of white masculine chivalry.^76^ If the explicitly white supremacist “Rape-Lynch Mythology” has faded from popular view, the traces of these tropes of gendered white precarity remain in the form of less recognizably racist appeals to white vulnerability from criminal strangers, which in turn feeds the proliferation of SYG laws. The facial neutrality of these laws veils their empowerment of *some* civilians — most often white, propertied, and masculine — to use deadly force against “strangers” they consider threatening. In other words, SYG laws are not simply a natural extension of the Castle Doctrine, as proponents insist, they are part of a package of modern policies that intensify the historic white supremacy, sexism, and settler colonialism embedded in our legal system and broader social structures.

## Next Steps in SYG Research

Our review of contemporary research reveals a rich variety of methods and approaches used to assess the relationship between gender and SYG laws. However, the efficacy, breadth, and inclusiveness of the available research can be improved. A robustly intersectional approach considers how gender, race, ethnicity, and class simultaneously influence the experience of self-defense generally as well as the specific adjudication of SYG laws. Future research must look beyond a traditional focus on white women to consider how existing legal structures disadvantage non-white, low-income, disabled, and queer women when it comes to (1) defending themselves from violence, especially from their own intimate partners, and (2) seeking redress through courts.^77^ Empirical studies should examine the impacts of SYG laws on the most common forms of violence against women, including domestic and sexual violence, by attending to the relationship between defendant/claimant and victim/alleged assailant. Moreover, we recommend rigorous studies using data from all states where SYG laws have been implemented to interrogate the differences in outcomes of cases by race, gender, relationship, and sexuality, especially within the context of DV and/or IPV.

Future research must also investigate how SYG laws evolved separately from legal strategies designed to protect women, such as BWS. Contrary to the rhetoric of conservative policymakers and “gun rights” advocates, SYG laws and self-defense laws more generally were never designed to protect women or to give them legal recourse when they resist violence from their abusers. Studies that interrogate the complex impact of SYG laws must adopt a more inclusive approach, in an effort to expose how biased assumptions are baked into our legal codes and practices, naturalizing legal double standards that incentivize violence for the privileged few while intensifying the precarity of the most vulnerable.

## Note

The authors have no conflicts to disclose.
